# Measuring management’s perspective of data quality in Pakistan’s Tuberculosis control programme: a test-based approach to identify data quality dimensions

**DOI:** 10.1186/s13104-018-3161-8

**Published:** 2018-01-16

**Authors:** Syed Mustafa Ali, Naveed Anjum, Maged N. Kamel Boulos, Muhammad Ishaq, Javariya Aamir, Ghulam Rasool Haider

**Affiliations:** 1 Monitoring, Evaluation and Learning Unit, Mercy Corps, Pak Palace, Rawal Chowk, Murree Road, Islamabad, Pakistan; 2 Alexander Graham Bell Center for Digital Health, University of Highlands and Islands, Inverness, Scotland UK

**Keywords:** Data quality, Data quality dimensions, Data quality improvement strategy, Informed decision-making, Test-based approach, Management perspective, Tuberculosis control programme

## Abstract

**Background:**

Data quality is core theme of programme’s performance assessment and many organizations do not have any data quality improvement strategy, wherein data quality dimensions and data quality assessment framework are important constituents. As there is limited published research about the data quality specifics that are relevant to the context of Pakistan’s Tuberculosis control programme, this study aims at identifying the applicable data quality dimensions by using the ‘fitness-for-purpose’ perspective.

**Results:**

Forty-two respondents pooled a total of 473 years of professional experience, out of which 223 years (47%) were in TB control related programmes. Based on the responses against 11 practical cases, adopted from the routine recording and reporting system of Pakistan’s TB control programme (real identities of patient were masked), completeness, accuracy, consistency, vagueness, uniqueness and timeliness are the applicable data quality dimensions relevant to the programme’s context, i.e. work settings and field of practice.

**Conclusion:**

Based on a ‘fitness-for-purpose’ approach to data quality, this study used a test-based approach to measure management’s perspective and identified data quality dimensions pertinent to the programme and country specific requirements. Implementation of a data quality improvement strategy and achieving enhanced data quality would greatly help organizations in promoting data use for informed decision making.

**Electronic supplementary material:**

The online version of this article (10.1186/s13104-018-3161-8) contains supplementary material, which is available to authorized users.

## Background

Public health services aim at preventing diseases and promoting health at the population level through the coordinated efforts of health authorities [1]. Planning or providing healthcare services involves either generation or consumption of healthcare data representing the health status of single individual or entire population [2]. Healthcare data and information are essential in assessing clinical encounters, in allocating resources, and in developing responsive health policies and practices and their monitoring [3]. Therefore, it is necessary to develop an approach for organizing information management processes and instituting the practices that will lead to data quality improvement [4].

In developing countries, the low performing health care delivery system and sub-optimal performance of healthcare providers have remained a serious concern [5]. Though use of routine data to formulate a targeted policy is a cost-effective approach of improving the public health care [6], most of the organizations have major data quality issues but they do not have any data quality improvement strategy [7]. Additionally, lack of consistent approach towards data, lack of professionalism and its standards are known to affect data quality [8].

Holistically, a successful data quality improvement strategy involves people, processes and technology. Defining roles and responsibilities of the data quality practitioners outlines the task-dependent processes (data collection, transfer, aggregation, etc.) contributing to the data quality improvement strategy [4]. However, the building units of a successful strategy must also consider the definition of data quality and the identification of data quality dimensions as well as their operational definitions and standards. Data quality is defined by two related objectives: (1) how well they fulfill the purpose of their intended use, and (2) how well they represent an object or event [9].

The benefit of having data quality improvement strategy, wherein data quality dimensions and data quality assessment framework are the important components of the strategy, is to have complete and correct data. In addition to ease in data collection, aggregation and analysis, data with improved quality will strengthen case-based reporting and TB surveillance system. Therefore, assessment of data quality and providing feedback to users are essential activities to generate an impact on TB control and prevention efforts [10]. National TB control programs of high-burden countries like Pakistan, Kenya, and Ethiopia have mentioned data quality issue as a serious concern implicating performance management negatively [11, 12]. However in TB control attempts, strategies for the data quality improvement are adopted elsewhere and have generated better data quality results [13]. The need for high quality data is also recognized by National Health Service (UK) and there is an agreement that all decisions, whether clinical, managerial or financial, are informed by the data of highest quality [8]. Essentially, data quality is improved to achieve better quality of care and performing healthcare system. Applying the ‘fitness-for-purpose’ perspective, quality refers to the extent to which data meet user requirements. The data quality literature provides an insight of the thorough cataloguing of data quality dimensions, whereas each dimension relates to a specific context of health practice and data management processes [14]. Moreover, a careful consideration of the complexity and realities of data management processes is essential for the meaningful assessment and improvement of data quality [15]. Therefore, a strong rationale for data quality assessment should be supported by the actions leading towards meeting user requirements and expectations [16].

Within Pakistan’s Tuberculosis control programme, technology adoption is relatively a recent activity, trying to meet an objective of making data available for the purposes of reporting and informed decision making quicker than before [17]. Hence, digital data collection offers an opportunity to receive data with better quality through programmed validation checks [18]. However, data quality in country’s context is less research subject and requires development of the data quality concept and quality improvement action plan. Meeting users’ expectations is the subjective aspect of data quality measurement [16], whereas, meeting specifications is the objective component [19].

Importantly, data quality is ensured when the perspective of the organization and its particulars are taken into account [20]. Data quality, being a context-specific concept, is not studied for Pakistan’s National TB Control program (NTP). Similarly, data quality dimensions, applicable to work settings of the Pakistan’s NTP are not identified before. The ‘fitness-for-purpose’ perspective helps knowing the intended use of the data and whether results of the data quality assessment will be accepted or not [21]. As there is limited published research about the data quality specifics that are relevant to the context of Pakistan’s Tuberculosis control programme, this study aims at identifying the applicable data quality dimensions by using the ‘fitness-for-purpose’ perspective.

## Methods

### Settings

According to the new funding model of the Global Fund, Mercy Corps Pakistan is the Principal Recipient (PR) of the grant for controlling Tuberculosis in 75 districts of the country. This grant supports the delivery of TB treatment services through a Public-Private Mix (PPM) model. Mercy Corps implements the programme through its seven partner organizations, which are called Sub-Recipients (SR). These SR organizations directly implement the TB control programme in their districts. Every quarter, a PR–SR coordination meeting is held to discuss the issues hampering or slowing down the progress of the programme, and at the same forum, immediate solutions are proposed for the programme’s smooth functioning. During the PR–SR coordination meeting held in September 2016, all participants—represented by the management of all SRs and Mercy Corps (PR)—were enrolled in the current study. Informed consent from the participants was taken.

### Data collection tool

A test-based approach was used to gather responses from the participants of the PR–SR coordination meeting. The format of the test was practical cases with multiple choices. All the questions were relevant to the field of practice (TB control) and specifics of the organization and management. Items of the test can be broadly categorized into: basic details of the respondent, knowledge about data and data quality, and practical cases of the data quality issues. Lead researcher and technical advisor for TB control programme reviewed the paper-based medical records independently and enlisted all practical cases. Using convenience sampling, practical cases of programmatic significance were selected for the test. For each question or practical case, relevant and confusing data quality dimensions were included as choices. It was pre-tested and revised before tis actual use. Importantly, no real identifiable patient details were used in the test.

### Data collection and analysis

During the PR–SR coordination meeting, all participants of the meeting were invited to participate in the research. Respondents were given the option to hide their identity and were allowed to take free time to complete the test. However, a response against each question was ascertained by the lead researcher.

In using test-based approach, respondents were provided multiple choice questions and were asked to select one option for each practical case. Response against each practical case is entered in Excel spreadsheet and frequencies were calculated. In case of selecting more than one option or missed response, respondent was asked to select one option to ensure quality of data. Highest frequency represented the application of ‘fitness-for-purpose’ perspective of the management. This way the data quality dimensions were identified for the data quality assessment framework.

## Findings

### Overview of respondents

42 personnel representing Mercy Corps and Sub-Recipient organizations were present at the PR–SR Coordination meeting; 19 personnel represented Principal Recipient unit of the Mercy Corps, and 23 personnel represented 7 Sub-Recipient organizations. These participants belonged to the different management levels, i.e., assistant, officer, coordinator and manager.

**Fig. 1:**
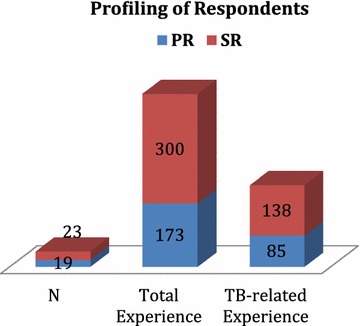
Profiling of respondents

The PR–SR coordination meeting pooled a total of 473 years of professional experience across participants, out of which 223 years (47%) were in TB control related programmes (Fig. 1). SR organizations alone had representation of 138 years of TB related experience (62%; 138 out of 223 years).

### Data: definition, use and quality

Respondents were asked about their general understanding of the data and their use. Forty-three percent of the respondents (18 out of 42) knew that text, numbers, and images all are forms of data. However, there was a majority (57%, 24 out of 42) who did not have a complete understanding of data. When asked “who defines the characteristics and standards of data quality”, only 38% (16 out of 42) of the participants considered “Data User” as a responsible person. Interestingly, 40% of the respondents (n = 17) considered data entry, information system, and monitoring and evaluation persons as the data users, probably because they are directly involved with data entry and processing tasks. Surprisingly, only 10% of the respondents (n = 4) described “complete and correct” as the comprehensive data quality logic. Seventy-one percent of the respondents (n = 30) did not know whether there is any data quality improvement strategy (DQIS) in place or not. However, 29% (n = 12) of the respondents said that there is a DQIS.

### Identification of the data quality dimensions

Respondents were presented with 11 practical cases or data quality issues that were selected from the local data recording and reporting formats. Choices included various data quality dimensions. The choice or dimension receiving most responses was to be considered for inclusion in the data quality assessment framework. However, after the test, the responses were discussed and an agreement was achieved among all the meeting’s participants.

### Completeness

The respondents described four practical cases (Table 1) as issues of ‘completeness’. The underlining concept matches the description of ‘completeness’, as proposed by World Health Organization [22], wherein a medical record is to be considered complete if it includes all related information with proper documentation. However, another description of ‘completeness’ is comprehensiveness of information whereby all required parts of an entity’s description are included [23].

**Table 1:** Practical cases reflecting the concept of ‘Completeness’

Practical cases	Percentage (%)	N
Number of patient records found in paper-based recording and reporting system does not match the number of records found on digital system	45	19
In the digital record of one patient, a valid data entry field is found vacant	79	33
Patient’s address is entered as Shehzad town	69	29
The contact number for patient is 0321413707	62	26

In one of the given practical cases (Table 1), Shehzad town as a patient’s address is considered incomplete by most of the respondents, because other important details (e.g., house, number, street number) are missed. Similarly, mobile contact number given in the case (0321413707) is considered as incomplete because a complete contact number contains 11 digits.

### Accuracy

There were three practical cases where the involved data quality issues were classified as ‘accuracy’ concerns. Respondents made their selection based on the knowledge of TB and its context. The understanding of the respondents was closely matched by the definition of ‘accuracy’ proposed by Pratiwi and Anawar [24]. According to them, an issue of accuracy exists when a measurement (or value) does not match the actual value.

According to operational guide of the PPM model, every district and registered healthcare provider is given a unique code. Hence, any deviation from the coding scheme is considered an issue of accuracy (Table 2). Similarly, first four digits of the contact number, presented in the case, are not in accordance with the national coding system (Table 2).

**Table 2:** Practical cases reflecting the concept of ‘Accuracy’

Practical cases	Percentage (%)	N
The Patient Identifier Number generated for Hafizabad-based patient is P-09-G002-142-16; however, the district code for Hafizabad is 11, not 09	57	24
Two identical Patient Identifier Numbers with same names were found in records. They were entered by two different General Practitioners	60	25
The contact number for patient is 01234137098	57	24

### Consistency

There was only one practical case which was identified by the management of TB control programme as an issue of ‘consistency’ (Table 3). According to Almutiry et al. [25], ‘consistency’ is achieved when “representation of data values remains the same in multiple data items in multiple locations”.

**Table 3:** Practical cases reflecting the concept of ‘Consistency’

Practical cases	Percentage (%)	N
Records show that Patient ABC is registered as pulmonary case, while on follow up visit his services are recorded as extra-pulmonary case	40	17

### Vagueness

One practical case was identified as an issue of ‘vagueness’ (Table 4). If a statement or the term is unclear or unspecific then it is regarded as vague [24].

**Table 4:** Practical cases reflecting the concept of ‘Vagueness’

Practical cases	Percentage (%)	N
One record shows patient name as Kaki (*considered ‘vague’ in Pakistan*)	64	27

### Duplicate or uniqueness

Seventy-four percent of the respondents (n = 31) described a duplicated record as the opposite of ‘uniqueness’. Batini et al. [26] also identified the number of duplicates as a ‘uniqueness’ concern. Only one instance among the practical cases was identified as a ‘uniqueness’ issue (Table 5).

**Table 5:** Practical cases reflecting the concept of ‘Uniqueness’

Practical cases	Percentage (%)	N
Two identical Patient Identifier Numbers with same patient names were found in the records; however, both records were entered by the same General Practitioner	64	27
The opposite to duplicate is___________	74	31

### Timeliness

Among the test items, there was one data quality issue which was identified as ‘timeliness’ concern by majority of respondents (Table 6). Orfanidis et al. [27] defined ‘timeliness’ as follows: “Shared data should be as near real-time as possible. Thus, data should be timely, in that it relates to the present”.

**Table 6:** Practical cases reflecting the concept of ‘Timeliness’

Practical cases	Percentage (%)	N
For digital records, if patient is registered on 19th August 2016 and registration data reaches server on 25th August 2016 (*within* 1 *week*)	86	36

## Discussion

The data quality dimensions, identified using test-based approach, are consistent with terminologies found in existing literature on data quality. However, the approach of collecting or identifying data quality dimensions in TB control programs implemented in other parts of the world is different, e.g., survey [7], qualitative interview [8], literature review [11] and consultative workshop [30]. In other domains and instances, business data consumers identified 179 data quality dimensions through survey [28], and a review of electronic medical record literature identified 27 data quality dimensions [29].

In our study, we used test-based approach to collect management’s perspective about the quality of TB related data, which has never been reported before in Pakistan. Data quality holds a central position in TB-related programmes around the world and similar approach is used elsewhere to detect and fix data quality issues [30]. Even one of the remarkable healthcare systems of the world, National Health Service (NHS), involved many stakeholder organizations to record their perception about data quality in the NHS [8]. In other businesses it has been acknowledged that operationalizing data quality without invoking the local perspective can be challenging [15].

Good quality data is a pre-requisite of data use, as it develops its users’ trust over the sanity of data [31]. Funding agencies like, US President’s Emergency Plan for AIDS Relief (PEPFAR) and the Global Fund (GF) have highlighted the importance of data quality assessments at healthcare facility level to ensure valid reports for key performance indicators of the programme coverage [32]. For instance, Global Fund provides program and data quality assessment guidelines because there is an increased focus on both the data quality and service delivery quality [33]. To support this objective, standard tools and processes for routine data quality audits of the primary data sources are developed. This approach is cost-intensive and is generally not feasible for healthcare facilities to use them on their own on regular basis to monitor data quality and its improvement [34].

Recently, GF has acknowledged the context specificity of the assessment tools and processes. Therefore, in revised approach of program and data quality assessment, Global Fund has moved from uniform assessment approach to tailored or country-specific approach. Similarly, GF suggests national processes and tools instead of using GF-specific processes and tools for program and data quality assessments [35].

It is already argued that identification of the data quality dimensions uses ‘fitness-for-purpose’ perspective and one such example is data quality assessment of the clinical data for the use of research, where completeness, accuracy and consistency were identified as applicable data quality dimensions [36]. Similarly, data quality assessment tools and approaches are developed which are specific to the field of practice within healthcare system.

## Conclusion and recommendation

Data quality is important for performance measurement and effective decision making. Based on a ‘fitness-for-purpose’ approach to data quality, this study used a test-based approach to measure management’s perspective and identified data quality dimensions that are most appropriate for the field of practice (Tuberculosis control) and work settings (Pakistan). Completeness, accuracy, consistency, vagueness, uniqueness and timeliness are the data quality dimensions relevant to the context of Pakistan’s Tuberculosis control programme. Data quality dimensions are central to the development of a data quality assessment framework and a data quality improvement strategy. Measuring management’s perspective of data quality is an effective way of learning about users’ expectations that are associated with the intended uses of the data. Implementation of a data quality improvement strategy and achieving enhanced data quality would greatly help organizations in promoting data use for informed decision making.

Though the literature discusses ‘fitness-for-purpose’ perspective as adherent idea to data quality concept, different approaches to collect management’s perspective on ‘fitness-for-purpose’ are not published. However, there is voluminous literature on data quality suggesting need for identification of data quality dimensions and data quality improvement strategy. This research proposes practical approach to collect data user’s requirements and to subsequently identify applicable data quality dimensions. Therefore, this work can serve as a model that can be readily adopted or easily adapted by other locales with similar low-resource settings. Future research should also focus on utilizing different approaches to identify applicable data quality dimensions according to the field of practice and work settings.

## Additional file

**Additional file 1.** Measuring Data Quality Perspective.

## Abbreviations

PR: Principal Recipient
SR: Sub-Recipient
TB: Tuberculosis
PPM: Public–Private Mix
DQ: data quality
DQIS: data quality improvement strategy
DQAF: data quality assessment framework
