# Supplementary material for: Measuring management’s perspective of data quality in Pakistan’s Tuberculosis control programme: a test-based approach to identify data quality dimensions
Source: BMC Res Notes. 2018 Jan 16;11:40. doi: 10.1186/s13104-018-3161-8 (PMC5771188; doi:10.1186/s13104-018-3161-8)
Supplement: Supplementary file 1 — Additional file 1. Measuring Data Quality Perspective. [file 13104_2018_3161_MOESM1_ESM.docx]

**Measuring Data Quality Perspective**

Name (Optional): ____________________________________________________________________________________

Management Level (SR/PR): _______________________________________________________________________

Total Experience (in years):_________________________________________________________________________

Total Experience in TB related health programs (in years): _____________________________________

***Note:*** Please select the most appropriate option bearing in mind that at any response is neither right nor wrong, but appropriate or inappropriate.

1. Data can be______;

| 1. Text | 1. Number |
| --- | --- |
| 1. Image | 1. a and b |
| 1. All of the above |  |

1. Who defines characteristics and standards of the data quality?

| 1. Program manager/officer | 1. M&E or Information System person |
| --- | --- |
| 1. Data entry person | 1. Patients and their family members |
| 1. Data Users |  |

1. Which phrase best describes “Data Quality”?

| 1. Fitness-for-reporting | 1. Fitness-for-use |
| --- | --- |
| 1. Fit-to-context | 1. Fit-to-donor requirement |

1. Which two basic dimensions complete data quality logic?

| 1. Precise and Accurate | 1. Complete and Accurate |
| --- | --- |
| 1. Complete and Correct | 1. Precise and Valid |

1. Which word best describe the situation illustrated below (Hint: Completeness, Accuracy, Validity, and Precision)

| 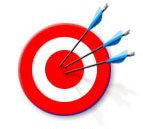 | 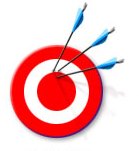 |
| --- | --- |
| Accuracy | Precision |

1. Number of patient records found in paper-based R&R system does not match the number of records found on digital system. Please select issue type

| 1. Timeliness | 1. Accuracy |
| --- | --- |
| 1. Completeness | 1. Consistency |

1. In digital record of one patient, a valid data entry field is found vacant. Please select issue type

| 1. Completeness | 1. Validity |
| --- | --- |
| 1. Accuracy | 1. Consistency |

1. The Patient Identifier Number generated for Hafizabad-based patient is **P-09-G002-142-16** however, the district code for Hafizabad is 11. Please select issue type

| 1. Completeness | 1. Validity |
| --- | --- |
| 1. Accuracy | 1. Consistency |

1. Records show that Patient ABC is registered as pulmonary case, while on follow up visit his services are recorded as extra-pulmonary case. Please select issue type;

| 1. Completeness | 1. Vagueness |
| --- | --- |
| 1. Accuracy | 1. Consistency |

1. One record shows patient name as Kaki. This is an issue of;

| 1. Completeness | 1. Vagueness |
| --- | --- |
| 1. Accuracy | 1. Consistency |

1. Two identical Patient Identifier Numbers with same names were found in records, however, entered by two different GPs;

| 1. Duplicate | 1. Vagueness |
| --- | --- |
| 1. Accuracy | 1. Consistency |

1. Two identical Patient Identifier Numbers with same names were found in records, however, entered by same GP is an issue of;

| 1. Duplicate | 1. Vagueness |
| --- | --- |
| 1. Accuracy | 1. Consistency |

1. Patient’s address is entered as Shehzad town, this is likely an issue of;

| 1. Vagueness | 1. Completeness |
| --- | --- |
| 1. Accuracy | 1. Consistency |

1. The contact number for patient is 01234137098. This is an issue of;

| 1. Vagueness | 1. Completeness |
| --- | --- |
| 1. Accuracy | 1. Consistency |

1. The contact number for patient is 0321413707. This is an issue of;

| 1. Vagueness | 1. Completeness |
| --- | --- |
| 1. Accuracy | 1. No Issue |

1. For digital records, if patient is registered on 19^th^ August 2016 and it reaches server on 25^th^ August 2016. This is an issue of;

| 1. Timeliness | 1. Accuracy |
| --- | --- |
| 1. Completeness | 1. Consistency |

1. The opposite to duplicate is;

| 1. Valid | 1. Timeliness |
| --- | --- |
| 1. Uniqueness | 1. Accurate |

1. What is our Data Quality Improvement Strategy (DQIS) in PPM?

| 1. Don’t know | 1. There is one |
| --- | --- |
| 1. There is no DQIS |  |
